# Hyperpolarized ^13^C metabolic imaging detects long-lasting metabolic alterations following mild repetitive traumatic brain injury

**DOI:** 10.21203/rs.3.rs-3166656/v1

**Published:** 2023-08-14

**Authors:** Myriam Chaumeil, Caroline Guglielmetti, Kai Qiao, Brice Tiret, Mustafa Ozen, Karen Krukowski, Amber Nolan, Maria Serena Paladini, Carlos Lopez, Susanna Rosi

**Affiliations:** University of California, San Francisco; University of California, San Francisco; University of California, San Francisco; University of California, San Francisco; Bay Area Institute of Science, Altos Labs; Bay Area Institute of Science, Altos Labs; University of Washington; Bay Area Institute of Science, Altos Labs; Bay Area Institute of Science, Altos Labs; Bay Area Institute of Science, Altos Labs

## Abstract

Career athletes, active military, and head trauma victims are at increased risk for mild repetitive traumatic brain injury (rTBI), a condition that contributes to the development of epilepsy and neurodegenerative diseases. Standard clinical imaging fails to identify rTBI-induced lesions, and novel non-invasive methods are needed. Here, we evaluated if hyperpolarized ^13^C magnetic resonance spectroscopic imaging (HP ^13^C MRSI) could detect long-lasting changes in brain metabolism 3.5 months post-injury in a rTBI mouse model. Our results show that this metabolic imaging approach can detect changes in cortical metabolism at that timepoint, whereas multimodal MR imaging did not detect any structural or contrast alterations. Using Machine Learning, we further show that HP ^13^C MRSI parameters can help classify rTBI vs. Sham and predict long-term rTBI-induced behavioral outcomes. Altogether, our study demonstrates the potential of metabolic imaging to improve detection, classification and outcome prediction of previously undetected rTBI.

## INTRODUCTION

Individuals subject to frequent concussions such as career athletes (e.g. football players, boxers), accidental head trauma victims, domestic abuse victims, or active military are some of the population at risk for mild repetitive traumatic brain injury (rTBI). Indeed, rTBI is being steadily recognized as a risk factor for the development of epilepsy and neurodegenerative diseases, particularly chronic traumatic encephalopathy (CTE)^1, 2, 3^. However, to date, non-invasive diagnostic biomarkers of rTBI are lacking. In particular, clinical computed tomography (CT) and magnetic resonance imaging (MRI), the standard imaging methods for trauma patients, are unable to detect rTBI-induced pathology^4, 5, 6^. This lack of imaging techniques hampers proper diagnosis and appropriate clinical care, as a result new approaches are critically needed.

To further our understanding of rTBI, several models were developed over the past years^7, 8^. The closed-head impact model of engineered rotational acceleration (CHIMERA) device was designed to deliver multiple subconcussive mild TBI in a controlled and reproducible manner^9^. CHIMERA-induced rTBI has been shown to lead to reproducible pathological and behavioral changes up to several months following impacts^10^, mirroring the long-term effects of rTBI seen in the clinic (review by McNamara *et al.*^11^). Only a few studies have investigated the use of magnetic resonance imaging (MRI) to detect CHIMERA-induced rTBI. Diffusion MRI, which is sensitive to water diffusion in tissue and changes in tissue microstructure, detected subtle differences in rTBI animals at 7 days following injury and in the optic tract, brachium of the superior colliculus, corpus callosum and hippocampus regions^12, 13^; however, long-lasting changes were not studied. T_2_-weighted MRI, which is sensitive to changes in tissue microstructure, edema, and myelination, did not detect signs of brain injury at 7 days and 40 days post-injury^10, 12^. All these studies are in line with clinical findings, and further highlight the need for more sensitive approaches to detect and monitor long-term brain changes after rTBI.

Metabolic impairment following TBI has been well documented in patients and animal models in the hours following TBI using ^13^C-labelled substrates infusion and metabolomics approaches (reviews by Jalloh, Dermers-Marcil, and Carpenter^14, 15, 16^). Notably, cerebral microdialysis studies have identified that the lactate / pyruvate ratio (Lac/Pyr) parameter is associated with poor outcome^17, 18^. However, as cerebral microdialysis is an invasive method, its use at chronic timepoints following TBI, or in closed head injury and concussion is not feasible. Hyperpolarized ^13^C magnetic resonance spectroscopic imaging (HP ^13^C MRSI) is a unique technology that allows to measure metabolic fluxes *in vivo*, and to compute lactate / pyruvate ratio values as well. HP ^13^C MRSI applications have been extensively described in the oncology field^19, 20^, and its use is emerging to probe brain metabolism in health and diseases^21^. HP ^13^C MRSI of [1-^13^C]pyruvate enables to monitor the conversion of this key metabolite into its product(s) [1-^13^C]lactate and/or [^13^C]bicarbonate in the brain, which provides unprecedented metabolic information^22, 23^. Prior studies of moderate TBI have shown changes in the HP ^13^C lactate / pyruvate ratio (HP ^13^C Lac/Pyr), and HP ^13^C bicarbonate / lactate ratio at early timepoints (4 hours up to 7 days) following injury in preclinical models and in patients^24, 25, 26^, demonstrating the potential value of this technique. Furthermore, recent studies have combined injection of HP [1-^13^C]pyruvate with HP [^13^C]urea, a metabolically inactive probe, to simultaneously evaluate the pyruvate to lactate flux and tissue perfusion, respectively^27, 28, 29, 30, 31^. However, it remains to be determined whether HP ^13^C MRSI of HP [1–^13^C]pyruvate might be useful to the detection of rTBI at chronic timepoints, and inform on potential mechanism of metabolic injury. Furthermore, co-injection of HP [1-^13^C]pyruvate and HP [^13^C]urea has never been tested in any TBI model or patient.

Here, we questioned if advanced imaging approaches that have never been applied to the study of rTBI could detect long-lasting alterations following rTBI in the CHIMERA model. In addition to the above described HP ^13^C MRSI of HP [1-^13^C]pyruvate and HP [^13^C]urea, we also evaluated Susceptibility-weighted imaging (SWI) and $T_{1}$ mapping. SWI is an MRI method particularly sensitive to iron that can inform on venous deoxygenated blood and iron deposition in tissue, and which has proven very valuable to identify microbleeds in TBI, but has not yet been investigated in the CHIMERA model^32, 33^. Recent reports have highlighted the potential of $T_{1}$ mapping to detect oxidative stress in the rodent brain^34^, and thus this technique holds great potential to detect the production of reactive oxygen species that may occur following diffuse axonal injury and inflammatory processes observed in the CHIMERA model^11^.

**Figure 1:**
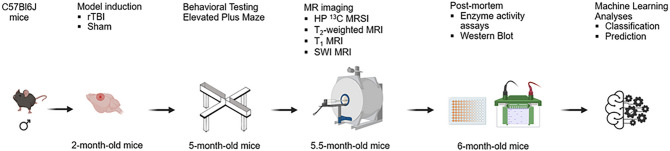
Study outline. Experimental timeline of the study. Two-month old male mice received a rTBI using the CHIMERA device or underwent a Sham procedure (no impact). Risk-taking behavior was evaluated at 3 months post-injury using the Elevated Plus Maze. MR imaging was performed 3.5 months after Sham or rTBI, and included HP ^13^C MRSI, T_2_-weighted MRI, $T_{1}$ mapping MRI, and SWI MRI. Tissue was collected 4 months after Sham or rTBI procedures to evaluate PDH and LDH activities, and expression of MCT1 and MCT4. ML analyses methods were used to identify the best classifiers between rTBI and Sham, and the best predictors of the risk-taking behavior and HP ^13^C Lac/Pyr in the cortex.

As shown in Fig. 1, we induced rTBI in two-month old male mice using the CHIMERA apparatus, tested risk-taking behavior 3 months post rTBI, and performed four MRI-based scans. We investigated the potential of ^13^C MRSI of HP [1-^13^C]pyruvate and [^13^C]urea to detect metabolic and tissue perfusion impairment, T_2_-weighted MRI to assess structural changes (as clinical standard of MR imaging), $T_{1}$-mapping to evaluate tissue microstructure alterations and oxidative stress, and SWI MRI to detect changes in tissue microstructure, microbleed and tissue oxygenation following rTBI. Last, brain tissue was collected to evaluate changes in enzymes activity and transporter protein expression. Given the multidimensional nature of the data, we used a Machine Learning (ML) approach to identify how measured parameters could best predict changes in risk-taking behavior and HP ^13^C MRSI.

## RESULTS

### HP ^13^C MRSI detects long-lasting metabolic alterations following rTBI

We investigated whether HP ^13^C MRSI could be used as a non-invasive tool to detect rTBI-induced long-lasting changes, and specifically to question whether metabolic alterations are present at chronic time points following injury.

**Figure 2:**
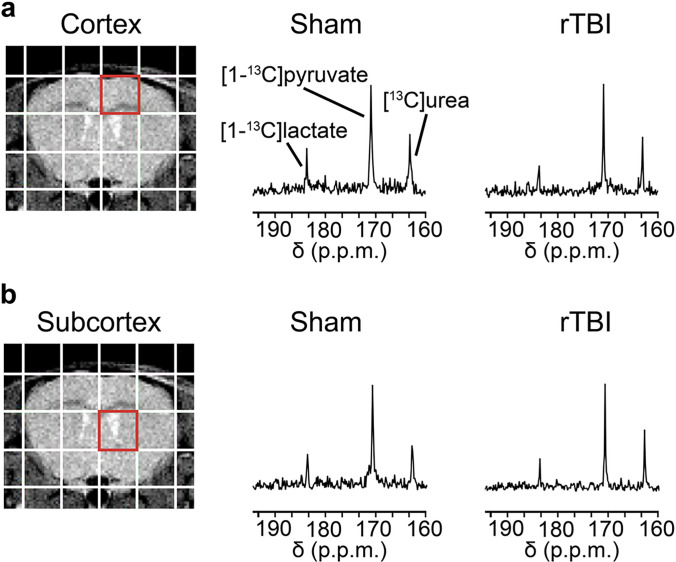
HP ^13^C spectra following co-injection of HP [1–^13^C]pyruvate and [^13^C]urea. Representative T_2_-weighted MR image overlaid with the grid used for HP ^13^C MRSI acquisitions. Representative ^13^C spectra showing HP [1-^13^C]pyruvate, HP [^13^C]urea and HP [1-^13^C]lactate in the **(a)** cortex (red voxel) and **(b)** subcortex (red voxel) for a Sham and a rTBI mouse.

**Figure 3:**
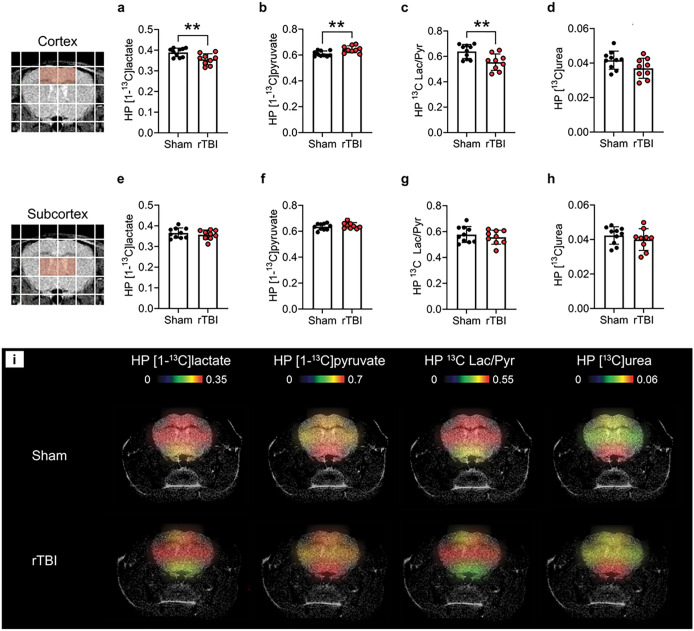
HP ^13^C MRSI detects long-lasting metabolic alterations following rTBI. Quantitative analyses of **(a)** HP [1-^13^C]lactate levels, **(b)** HP [1-^13^C]pyruvate levels, **(c)** HP ^13^C Lac/Pyr, and **(d)** HP [^13^C]urea for the cortex (highlighted red voxels), revealed lower HP [1-^13^C]lactate levels (p = 0.0073), higher HP [1-^13^C]pyruvate levels (p = 0.0073), and lower HP ^13^C Lac/Pyr (p = 0.0071) in rTBI compared to Sham mice. In contrast, quantitative analyses of **(e)** HP [1-^13^C]lactate levels, **(f)** HP [1–^13^C]pyruvate levels, **(g)** HP ^13^C Lac/Pyr, and **(h)** HP [^13^C]urea for the subcortex (highlighted red voxels), did not detect differences between rTBI and Sham mice. **(i)** Representative HP ^13^C heatmaps for a Sham and a rTBI mouse, highlighting lower HP [1-^13^C]lactate, higher HP [1-^13^C]pyruvate and lower HP ^13^C Lac/Pyr in cortical areas in rTBI mice. *N* = 9 rTBI and 10 Sham mice. Unpaired *t*-test (**p ≤ 0.01); data are expressed as means ± SD.

Following intravenous co-injection of HP [1-^13^C]pyruvate and HP [^13^C]urea, we observed signals from both substrates, as well as from the metabolic product HP [1-^13^C]lactate in the cortex (Fig. 2.a) and subcortex (Fig. 2.b) of Sham and rTBI mice at 3.5 months post-injury. Upon quantification, we observed significant metabolic differences between the cortex of rTBI compared to Sham mice: HP [1-^13^C]lactate levels were 1.09 fold lower in rTBI (Fig. 3.a, p = 0.0073), HP [1-^13^C]pyruvate levels were 1.05 fold higher (Fig. 3.b, p = 0.0073), and HP ^13^C Lac/Pyr was 1.15 fold lower (Fig. 3.c, p = 0.0071). No significant differences in HP [^13^C]urea levels were observed in the cortical area (Fig. 3.c), although a trend towards lower HP [^13^C]urea levels in rTBI was observed (p = 0.0794). In subcortical areas, we did not detect any differences in HP [1–^13^C]lactate, HP [1-^13^C]pyruvate, HP ^13^C Lac/Pyr and HP [^13^C]urea between rTBI and Sham mice (Fig. 3.e–h). In agreement with the metabolic quantifications, HP ^13^C heatmaps clearly show lower HP [1–^13^C]lactate, higher HP [1-^13^C]pyruvate and lower HP ^13^C Lac/Pyr in the cortex of rTBI mice (Fig. 3.i). Altogether, our results indicate that HP ^13^C MRSI can detect region-specific long-lasting metabolic changes following mild rTBI.

### Multimodal MRI does not detect long-lasting effect of injury in rTBI

We evaluated whether a comprehensive multimodal MRI approach could detect changes between rTBI and Sham mice at 3.5 months post-injury, when metabolic alterations where detected by HP ^13^C MRSI.

**Figure 4:**
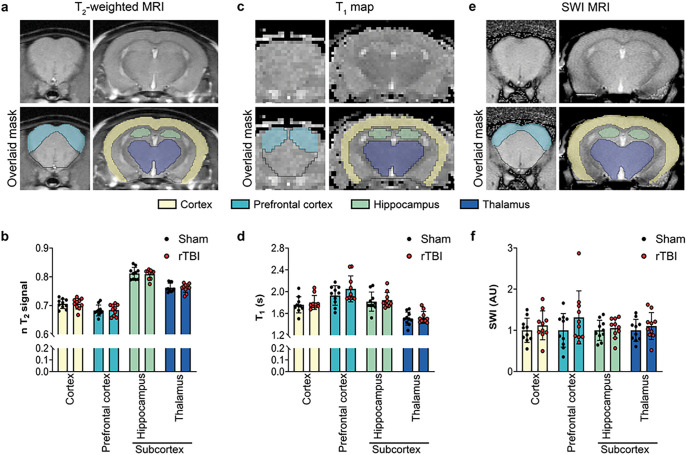
Multimodal MRI does not detect long-lasting effect of injury in rTBI. **(a)** Representative T_2_-weighted MRI data and corresponding manual brain masking. **(b)** Quantitative analyses of T_2_-weighted signal intensity revealed no significant differences for brain subregions between Sham and rTBI. **(c)** Representative $T_{1}$ map and corresponding manual brain masking. **(d)** Quantitative analyses of $T_{1}$ maps revealed no significant differences for brain subregions between Sham and rTBI. **(e)** Representative SWI data and corresponding manual brain masking. **(f)** Quantitative analyses of SWI intensity revealed no significant differences for brain subregions between Sham and rTBI. Brain masking color code: yellow: cortex, green: light blue: prefrontal cortex; hippocampus; dark blue: thalamus. *N* = 10 rTBI and 10 Sham mice. Unpaired *t*-test; data are expressed as means ± SD.

We first used T_2_-weighted MRI, the clinical standard of MRI approach, that is sensitive to inflammation and/or changes in myelin content. We found that T_2_ signal intensities were not different between rTBI and Sham mice in any of the regions studied (prefrontal cortex, cortex, hippocampus and thalamus (subcortex)) (Fig. 4.a–b). In addition, we did not detect any differences in brain region volumes between groups (**Supplementary Fig. 1**). Next, we used a $T_{1}$ mapping sequence that was shown to be sensitive to changes in microstructure or alterations related to oxidative stress. Similar to the T_2_ intensities, we did not observed any differences in the $T_{1}$ values in any of the region studied between rTBI and Sham mice (Fig. 4.c–d). Lastly, a SWI sequence was used, as it is capable of detecting microbleeds as well as potential changes in oxygenation following injury. Once again, no differences in SWI values were observed between rTBI and Sham mice in any region (Fig. 4.e–f). We did not detect any microbleed lesions in any of the studied animals.

Altogether, our results indicate that a comprehensive multimodal MRI approach combining T_2_-weighted MRI, $T_{1}$ mapping and SWI was not able to detect any signs of injury in rTBI mice at 3.5 months post-injury, unlike HP ^13^C MRSI.

### Disrupted enzymatic activity, but not transporter expression, is observed at chronic time points after rTBI

To further investigate potential underlying mechanisms responsible for the observed changes in HP ^13^C MRSI readouts, we evaluated the activity of enzymes responsible for pyruvate conversion into its downstream metabolites and the expression of transporters that control the entry of pyruvate into cells and the efflux of metabolites outside of the cells.

In the brain, lactate dehydrogenase (LDH) converts pyruvate into lactate, and pyruvate dehydrogenase (PDH) controls pyruvate entry into the tricarboxylic cycle and its conversion into acetyl-coA. We observed that PDH was 1.6 fold lower in the prefrontal cortex and 1.7 fold lower in the cortex of rTBI compared to Sham mice (Table 1, p = 0.0044 and p = 0.0375, respectively). No differences in PDH were observed in subcortical areas that include the hippocampus and thalamus. The activity of LDH was not significantly different between rTBI and Sham mice for cortical and subcortical areas.

As levels of HP metabolites and HP ^13^C Lac/Pyr also depend on HP [1-^13^C]pyruvate intake by cells and on the efflux of HP [1-^13^C]lactate, we evaluated the expression of monocarboxylate transporters (MCTs). MCT1 is primarily responsible for pyruvate entry into the cell, and MCT4 is principaly involved in the efflux of lactate outside of the cell. Protein quantification of MCT1 and MCT4 performed for the prefrontal cortex, cortex, hippocampus and thalamus did not show any differences between rTBI and Sham mice (Table 2), indicating that the differences observed with HP ^13^C MRSI are likely not due to MCT1 and MCT4 expression.

Altogether, our results indicate that PDH activity is decreased in the cortical areas following rTBI, while LDH activity, MCT1 and MCT4 expression are not significantly different compared to Sham at 3.5 months post-injury.

### Machine learning identifies rTBI/Sham classifiers, and predictors of behavior and HP ^13^C readouts

The machine learning (ML) analysis included all the data described above, as well as behavioral data we previously reported in Krukowski *et al.*^35^, which showed that mild rTBI leads to increased risk-taking behavior in male mice at 100 days post-injury.

Given n = 20 (10 for rTBI and 10 for Sham) mice along with 44 measured variables (see Table 3 for list of variables and abbreviations), we used ML to perform two types of analyses. First, we wanted to identify the best classifying variables allowing for separation of the two groups (rTBI vs Sham). Second, we aimed to find the best predictors of changes in risk-taking behavior, as it recapitulates a key behavioral component observed in rTBI patients, and of cortical HP ^13^C Lac/Pyr, due to its potential to serve as a novel biomarker for long-lasting consequences of rTBI.

**Figure 5:**
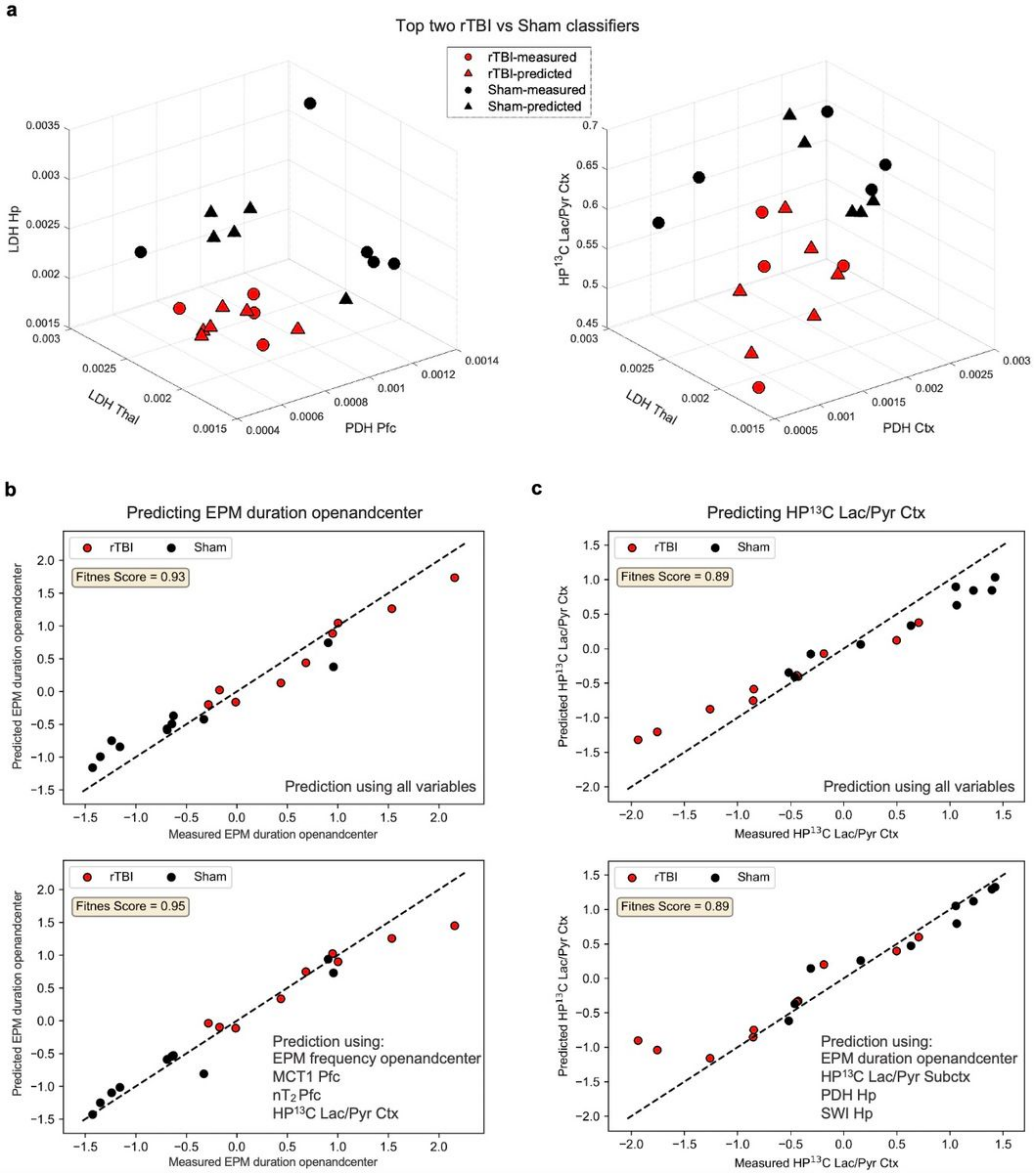
ML analyses identify best rTBI and Sham classifiers and best predictors of changes in risk-taking behavior and HP ^13^C Lac/Pyr Ctx. **(a)** Top two triplets that can classify rTBI (red) and Sham (black) mice. Here, circles represent the mice for which all three variables are measured whereas triangles represent mice for which at least one of the three variables were missing and predicted by ML algorithms (see Methods). **(b-c)** Prediction performance of the best predictors of risk-taking behavior (**b**, bottom panel), and HP ^13^C Lac/Pyr Ctx (**c**, bottom panel) compared to the prediction performance of the case in which all variables are used (**b-c** top panel). *N* = 10 rTBI and 10 Sham mice.

Various classification and feature extraction methods were implemented to identify the best classifying variables between Sham and rTBI mice. Consequently, we identified five triplets of variables that could accurately distinguish between either group and ranked them based on their feature importance scores computed using various feature extraction algorithms (see methods). The top two triplets with high feature scores are presented in Fig. 5.a and the others are shown in **Supplementary Fig. 2.** The top two triplets are: 1) PDH Pfc, LDH Thal, and LDH Hp, and 2) PDH Ctx, LDH Thal, and HP ^13^C Lac/Pyr Ctx. These findings suggest that although one single feature is not sufficient to identify the difference between rTBI and Sham, the combined PDH and LDH activity, as well as HP ^13^C imaging readouts can help distinguish differences between the two conditions. The three lower-tier triplets consisted of the subsequent variables: 3) PDH Pfc, EPM duration openandcenter, and LDH Thal, 4) PDH Pfc, EPM duration openandcenter, and HP ^13^C Lac/Pyr Ctx, and 5) PDH Pfc, MCT1 Pfc, and HP ^13^C Lac/Pyr Ctx. These three last triplets further idenfy the risk-taking behavior and MCT1 expression in the prefrontal cortex as important variables to classify Sham and rTBI.

Next, we identified the best predictors of the changes in risk-taking behavior and HP ^13^C Lac/Pyr Ctx presented in Fig. 5.b and 5.c, respectively. Interestingly, four variables, namely EPM frequency openandcenter, MCT1 Pfc, nT_2_ Pfc, and HP ^13^C Lac/Pyr Ctx, were sufficient to predict risk-taking behavior (Fig. 5.b bottom panel) with similar accuracy than when all variables were used for prediction (Fig. 5.b top panel). This result shows that a systems approach comprising behavioral, transporter expression, structural and HP ^13^C imaging measures yields a tangible prediction for changes in risk-taking behavior. Similarly, four variables, namely EPM duration openandcenter, HP ^13^C Lac/Pyr Subctx, PDH Hp, and SWI Hp, were enough to predict the HP ^13^C Lac/Pyr Ctx (Fig. 5.c bottom panel) with similar accuracy than when all the variables were used for prediction (Fig. 5.c top panel). This result demonstrates that using a systemic combination of behavioral, enzyme activity, and advanced MRI measures best predicts the HP ^13^C Lac/Pyr Ctx.

## DISCUSSION

In this study, we demonstrated that HP ^13^C MRSI of [1-^13^C]pyruvate can detect metabolic changes 3.5 months following rTBI when structural T_2_ MRI, $T_{1}$ mapping, SWI MRI and HP ^13^C MRSI of [^13^C]urea did not. Specifically, we measured a significantly lower HP ^13^C Lac/Pyr in the mouse cortex 3.5 months post-injury, which was associated with lower PDH activity. Using a ML approach, we further validated that the HP ^13^C Lac/Pyr is among the best classifiers of rTBI and Sham groups, and is a predictor of the risk-taking behavior observed in this rTBI model. Our findings demonstrate the ability of HP ^13^C MRSI of [1–^13^C]pyruvate to detect rTBI-induced damages and highlight promising potential to improve diagnosis and monitoring of rTBI patients at chronic time points when other imaging techniques are insufficient.

Cerebral metabolism has been probed using HP [1-^13^C]pyruvate both in preclinical and clinical settings in healthy and diseased brain (see Le Page & al. for recent literature review^21^). Prior studies performed in moderate TBI preclinical models of contusion injury have reported increased HP ^13^C Lac/Pyr at early timepoints following injury (4 hours up to 7 days following injury), but no significant changes at chronic timepoints (28 days post-injury)^24, 25^. In contrast, in this study we observed a decreased HP ^13^C Lac/Pyr at chronic timepoints, suggesting different underlying pathological changes between contusion injury and rTBI. Studies performed using positron emission tomography (PET) imaging with the glucose analogue ^18^F-fluorodeoxyglucose (^18^F-FDG) have detected long-term brain hypometabolism following TBI^4, 5, 36^, which is in line with the lower HP ^13^C Lac/Pyr measured here at chronic timepoints. To the best of our knowledge, ^18^F-FDG PET imaging has never been applied to CHIMERA rTBI model. Furthermore, the use of this method for TBI is limited by ionizing radiations, and the high background of ^18^F-FDG PET signal in the brain tissue, hampering the detection of small changes in glucose uptake.

Decreased HP ^13^C bicarbonate / lactate ratio was found in moderate TBI models^25^, and decreased HP ^13^C bicarbonate levels were found in TBI patients^26^, highlighting possible changes in mitochondrial function and aerobic versus anaerobic respiration following trauma. In agreement with these findings, a decreased of PDH activity after injury has been previously described^24, 37^, including in our current findings in rTBI. In this study, we were not able to detect ^13^C bicarbonate, likely due to the low signal to noise and the fast $T_{1}$ relaxation rate of this metabolite at ultra-high field (14.1 Tesla). HP ^13^C Lac/Pyr can be influenced by the activity of LDH and/or PDH, the availability of their cofactors^38, 39, 40^, as well as by MCTs expression, where increased MCT1 expression leads to increased HP ^13^C Lac/Pyr in selected cell lines^41^. In this study, we did not find any significant differences in MCT1 and MCT4 between rTBI and Sham mice, suggesting that they do not play a prominent role in the changes observed in the HP ^13^C Lac/Pyr at this late time post injury.

It has previously been shown that the blood-brain-barrier (BBB) limits the entry of HP probes, which could in turn influence the measured HP ^13^C Lac/Pyr^42, 43^. To evaluate potential changes in perfusion and delivery, we co-injected [1-^13^C]pyruvate with HP [^13^C]urea, a metabolically inactive probe. Although decreased cerebral blood flow alteration has been reported following rTBI^44, 45^, we did not observe any differences in HP [^13^C]urea levels between Sham and rTBI, indicating that perfusion was likely unaltered at 3.5 months post-injury. To the best of our knowledge, this is the first study reporting the use of HP [^13^C]urea in a rTBI model. Additional studies at earlier timepoints following injury, and in other TBI models, are needed to evaluate the full potential of HP [^13^C]urea in detecting BBB alterations and/or vasculature changes following brain injury.

Conventional and advanced anatomical MRI did not detect any differences between Sham and rTBI at 3.5 months post-injury. In agreement with these findings, Haber *et al.* and our group previously reported no differences using T_2_ MRI at 7 days and 40 days post-injury, respectively^10, 12^. These results suggest that conventional T_2_ MRI may not be able to detect rTBI patholology-induced using the CHIMERA device, either at early or late timepoints following injury. Using $T_{1}$ mapping we investigated whether we could detect changes in tissue microstructure and reactive oxygen species production, but found no differences between Sham and rTBI^34^. Oxidative stress and reactive oxygen species have been shown to play an important role in TBI pathogenesis and in mediating axonal degeneration^46, 47, 48^. However it remains unclear if these events may predominantly occur at early timepoints following injury and would have resolved by the time we performed our imaging study (3.5 months injury), or whether $T_{1}$ mapping was not able to detect these events in this rTBI model. HP [1-^13^C]dehydroascorbic acid (DHA) and HP [1-^13^C]N-acetyl cysteine (NAC) have been shown to be sensitive probes to investigate redox changes *in vivo*^49, 50^, and therefore represent attractive probes to further interrogate the involvement of oxidative stress using HP ^13^C MRSI. We also included SWI MRI exams as this method has been shown to improve the detection of microbleeds and hemorrhagic diffusive axonal injury after TBI, which was associated with neurologic deficits and long-term outcome in human TBI^51, 52^. However, we did not detect any differences in SWI MRI between Sham and rTBI. As for $T_{1}$ mapping, it remains to be determined whether no microbleeds or oxygenation changes occurs in this model at early timepoints, or whether potential changes have resolved by the time we conducted our imaging exams.

We took a systems approach to measure behavior outcomes using ML analyses. We found that sets of variables were able to classify rTBI and Sham mice. These variables are linked to cognitive abilities (risk-taking behavior), metabolism and molecule transport (PDH and LDH activity, HP ^13^C Lac/Pyr, and MCT1), highlighting the importance of long-term metabolic impairment in rTBI and suggesting their potential as injury biomarkers. Interestingly, ML was able to identify that enzymatic changes in the thalamus and hippocampus regions, which were not statistically significant using conventional unpaired t-test in isolation, became important variables to classify rTBI and Sham when considered together. This is in agreement with changes in hippocampal function that have been previously reported in rTBI up to 6 months post-injury^53^. Future HP ^13^C imaging studies will aim to also include the thalamus and hippocampus to determine whether *in vivo* metabolic changes can be detected in these regions. ML was also used to determine which variables are best predictors of the risk-taking behavior. We found that the changes in risk-taking behavior were best predicted by variables from the cortical areas, including HP ^13^C Lac/Pyr, MCT1 expression, structural MRI, and behavioral parameters. Similarly, cerebral microdialysis studies have shown that the Lac/Pyr is an important variable associated to clinical outcome. High Lac/Pyr within the first days after injury was associated to poor clinical outcome 6 months later^18^, while here we show that a lower HP ^13^C Lac/Pyr is associated to higher risk-taking behavior 3.5 months after injury. This discrepancy might be explained by different timing of the Lac/Pyr measurement (days versus months after injury), and the injury severity (severe TBI versus mild rTBI). Nonetheless, our study provides further evidence that the Lac/Pyr is a useful marker to predict behavioral outcome, which can now be measured in a non-invasive manner using HP ^13^C MRSI, thus opening new avenues to evaluate metabolic alterations months after trauma. Last, ML was used to determine the best predictors of the HP ^13^C Lac/Pyr in the cortex, and identified subcortical variables, including PDH activity, SWI MRI and metabolic MRSI, as well as risk-taking behavior. Altogether, these results demonstrate the importance of multimodal approaches to detect rTBI pathology and associated long-lasting changes.

Thanks to the recent efforts of the community to enable easy data sharing though data repository^54^, future studies with higher sample size will become possible, which in turn can lead to improvement of our understanding of biological and functional pathway involved in rTBI, and help identify novel biomarkers.

In summary, our findings demonstrate the potential of HP [1-^13^C]pyruvate to detect long-lasting metabolic alterations in a mouse model of rTBI. In addition, ML identified HP ^13^C MRSI as a key parameter to predict long-term rTBI-induced behavioral outcomes. Over the past few years, the use of HP ^13^C MRSI in clinical trials worldwide has been rapidly expanding, and the injection of HP [1-^13^C]pyruvate has proven feasible and safe, with no reported side effects^55^. In this study, we were able to measure changes in HP ^13^C MRS parameters from two regions, the cortex which is closest to the impact, and the subcortex, which is more remote. We were not able to differentiate between smaller brain regions (e.g. prefrontal cortex, hippocampus, thalamus) due to the large voxel size used in this study relative to the size of the mouse brain. Current sequences available on clinical scanners can achieve up to 1 cm^3^ spatial resolution and cover the entire human brain, thus providing metabolic information from brain areas close and remote to the site of injury. With the growing availability of the HP ^13^C MRS technology, our findings provide a strong rationale to translate its use in patients suffering from rTBI, with the aim to improve the detection of rTBI-induced damages, help in understanding metabolic pathways involved in rTBI pathogenesis, and eventually aid the development of treatment strategies.

## METHODS

### Animals and rTBI model induction

All animal research was approved by the Institutional Animal Care and Use Committee of the University of California, San Francisco. Mice were given one week of acclimation and housed with a reversed 12-h light/12-h dark cycle and provided food and water ad libitum. At 8 weeks of age, mice were randomly assigned to the rTBI or sham control group. Animals were anesthetized using isoflurane (2–3%) in oxygen 1 L/min during the procedure. rTBI animals were subjected to multiple, mild, closed-head injuries using the CHIMERA device as previously reported^10, 35^. Briefly, rTBI animals were placed supinely into an angled holding platform without any shaving of the head or incision into the skin so that the head was level with the piston target hole while aligning the eyes, ears, and nose such that the impact was centered on the dorsal convexity of the skull, targeting a 5-mm area surrounding bregma. A nose cone delivering isoflurane was removed just prior to the impact. Impact was initiated using RealTerm software, which was connected to a system including air tank, pressure regulator, digital pressure gauge, two-way solenoid valve, and piston. The impact was administered with a velocity range of 3.9–4.5 m/sec, resulting in an impact energy of 0.5 J from the 5 mm, 50 g piston^10, 35^. Animals were moved to an incubator immediately after the impact and monitored until fully recovered. rTBI animals received an injury once per day for 5 days with a 24 h interval in between impacts. Five repeated hits were chosen to specifically focus on the effects of repeated exposure to TBI, as athletes, veterans and sometimes trauma victims are exposed to constant and repeated blows, even without experiencing concussive symptoms. Sham mice were exposed to the same isoflurane anesthesia paradigm without sustaining an impact. Skull fractures, seizures, apnea, or mortality were not observed in any animals, and no animals were excluded from the study due to injury parameters.

### Risk-taking behavioral test

For all behavioral assays, the experimenters were blinded to surgery. Before behavioral analysis, animals were inspected for gross motor impairments. Animals were inspected for whisker loss, limb immobility (including grip strength), and eye occlusions. If animals displayed any of these impairments, they were eliminated from the study. Behavioral tests were recorded and scored using a video tracking and analysis setup (Ethovision XT 8.5, Noldus Information Technology). If tracking was unsuccessful, videos were scored by two individuals blinded to surgery. Risk-taking behavioral phenotype was evaluated using the Elevated Plus Maze (EPM) at ~ 100 days (3 months) post-injury (counted from the day of the first injury) as described previously^10, 35^. The EPM consists of two exposed, open arms (35 cm) opposite each other and two enclosed arms (30.5 cm) also across from each other. The four arms are attached to a center platform (4.5 cm square), and the entire maze is elevated 40 cm off the floor. Bright white lights illuminated both ends of the open arm. Mice were placed individually onto the center of the maze and allowed to explore the maze for 5 min, and their activity was recorded. The maze was cleaned with 70% ethanol between animals. Risk-taking behavior was measured by changes in time spent in the open arms + center of the EPM. We also measured the number of entries into the extremes zones on the EPM, the time spent in the extreme zons on the EPM, the number of entries in the open arms + center on the EPM, the total distance traveled on EPM measured by centerpoint, and the average animal velocity on the EPM. These behavioral data were previously reported in Krukowski *et al.*^35^.

### Magnetic resonance imaging

Mice were anesthetized using isoflurane (1.5–2% in O_2_) and a 27-gauge catheter was placed in the tail vein to allow for intravenous (i.v.) injection. Next, animals were placed in a dedicated cylindrical cradle allowing for reproducible positioning of the mouse head; which was subsequently inserted inside a dual tune ^1^H-^13^C volume coil (ØI = 40 mm) or a single tuned ^1^H proton coil (ØI = 40 mm) in a 14.1 T vertical MR system (Agilent Technologies). Respiration rate was continuously monitored through the PC-sam software interface (SA Instrument, NY, USA).

First, T_2_-weighted images from the entire brain were acquired for adequate positioning of the grid used for hyperpolarized ^13^C acquisitions using the following parameters: repetition time (TR) = 1200 ms, echo time (TE) = 20 ms, slice thickness 1.8 mm, 2 averages, matrix 256 × 256, field of view (FOV) 30 × 30 mm². For HP ^13^C MRSI acquisitions, 24 μL of [1-^13^C]pyruvate and 55 μL [^13^C]urea preparation were co-polarized using a Hypersense DNP polarizer (Oxford Instruments) for one hour. After dissolution, the HP [1–^13^C]pyruvate and [1-^13^C]urea preparation was rapidly dissolved in isotonic buffer (pH ~ 7) to a final concentration of 80 mM and 78 mM, respectively. A final volume of 300 μL of the HP [1-^13^C]pyruvate and [^13^C]urea solution was then injected i.v. through the tail vein catheter. 2D dynamic chemical shift imaging ^13^C data were acquired 16 seconds post i.v. injection of the HP [1-^13^C]pyruvate and [^13^C]urea solution using the following parameters: TR = 67 ms, TE = 0.58 ms, spectral width 5000 Hz, 256 points, flip angle 10°; matrix = 16 ×16, field of view (FOV) = 32 × 32 mm²; slice thickness 4 mm.

Next, for T_2_-weighted MRI, $T_{1}$ MRI and SWI acquistions, the dual tune ^1^H-^13^C volume coil (ØI = 40 mm) was removed and replaced by a ^1^H volume only coil (ØI = 40 mm). T_2_-weighted MRI was acquired using a 2D fast spin-echo, with effective echo time (TEeff)/TR = 11.80/2006 ms, FOV = 25 × 25 mm^2^, in 256 × 256 array and 0.5 mm slice thickness. $T_{1}$-mapping data were acquired using fast spin-echo with inversion recovery: TEeff/ TR = 7.44/10000 ms, 8 inversion times (TI): 100, 170, 310, 530, 940, 1640, 2900, 5000 ms, FOV = 30 × 30 mm^2^, in 128 × 128 array and 1 mm slice thickness. SWI acquisitions were acquired using TEeff/TR = 4.64/111.29 ms, FOV = 20 × 20 mm^2^, in a 256 × 256 array and 0.4 mm slice thickness.

### Magnetic resonance imaging data analysis

Brain regions were manually delineated on $T_{1}$ maps, T_2_-weighted and SWI magnitude images for each mouse based on the Allen Adult Mouse Brain atlas (Allen Institute) using the Aedes region of interest package for MATLAB (Mathworks). For each region, brain volumes were calculated with the T_2_-weighted data, as well as the mean T_2_-weighted values which were normalized to the mean of the cerebrospinal fluid signal from the ventricles as signal value standard. The mean $T_{1}$ relaxation times was calculated from $T_{1}$ maps generated in VNMRj by pixel-wise fitting according to (Eq. 1).

(eq. 1) $$y=|\left(M(0)-M_{0}\right)\;*\;e^{\left(\frac{t}{T1}\right)}+M_{0}|$$

Where y is the measured signal from fast spin echo with multiple inversion recovery, and the three fit parameters: relaxation time $T_{1}$, equilibrium longitudinal magnetization $M_{0}$, and pre-inversion recovery longitudinal relaxation M(0). The TI list is used as input for time t.

SWI data were processed as previously described^32^ with phase images unwrapped by PRELUDE (FSL), High pass Gaussian filtered with pixel size 32 × 32, and positive phase map scaling used (Eq. 2).

(eq. 2) $$\varphi_{pos}(t)=\left\{\begin{array}{cc} \frac{\left|\varphi_{max}\right|\;-\;\varphi (t)}{\left|\varphi_{max}\right|} & \varphi (t)>0\\ 1 & \varphi (t)<0 \end{array}\right.$$

Normalized positive phase map $\varphi_{\text{pos}}(t)$ where φ(t) is the filtered, unwrapped phase at location t, and $\varphi_{max}$ is the maximum phase of the slice of interest. The positive phase map is a spatial map varying between zero and one, with higher phase approaching zero and thus increasing contrast on the final merged SWI data. The phase map is multiplied with the magnitude image four times to create final SWI data^56^. The mean SWI intensity was calculated for each mouse for each brain region and normalized to the mean of the Sham for each region, which corresponds to 1.

HP ^13^C MRS imaging data was analyzed using the in-house SIVIC software (http://sourceforge.net/apps/trac/sivic/) and custom-built programs written in MATLAB (MATLAB R2011b, The MathWorks Inc.). The area under the curve (AUC) of HP [1-^13^C]pyruvate, AUC of HP [1–^13^C]lactate and AUC of HP [^13^C]urea were measured for each voxel and normalized to the noise level. To account for variations in polarization levels and delivery, HP [1-^13^C]pyruvate signal and HP [1-^13^C]lactate signal were calculated by dividing each value by the sum of HP [1-^13^C]pyruvate and HP [1-^13^C]lactate for each voxel. HP [^13^C]urea signal was obtained by normalizing HP [^13^C]urea signal from each brain voxel to the sum of [^13^C]urea signal from tissue surrounding the brain. HP ^13^C Lac/Pyr was calculated as the ratio of the AUC. Next, average from voxels containing cortex or subcortex was calculated, and the obtained mean values were used to evaluate statistical significance between sham and rTBI groups. Color heatmaps of HP [1-^13^C]pyruvate, HP [1-^13^C]lactate, HP [^13^C]urea and HP ^13^C Lac/Pyr were generated using a linear-based interpolation of the ^13^C 2D CSI data to the resolution of the anatomical images using custom-built programs written in MATLAB and SIVIC.

### Ex vivo analyses of brain samples

Following the imaging session, mice were transcardially perfused with ice-cold phosphate buffered saline, and brains were rapidly dissected. Next, prefrontal cortex, cortex, thalamus and hippocampus were isolated and snap-frozen in liquid nitrogen. Samples were stored at − 80°C until further processing for activity assays and western blots.

LDH and PDH activities were evaluated using spectrophotometric activity assay kits according to manufacturer’s guidelines (ab102526 and ab109902; Abcam, respectively), and normalized to protein concentration determined by the Bradford protein assay method.

For western blot analyses, frozen brain samples were then homogenized with RIPA buffer (Pierce, 89900) and protease inhibitor Halt ^™^ Protease Inhibitor Cocktail (Thermo Scientific, 1862209) using a TissueLyser II (Qiagen). Lysate were incubated on ice for 15 minutes and then centrifuged at 14,000 rpm for 10 min at 4°C. Protein concentration in supernatants was determined using the Bradford protein assay method. Equal amount of proteins was loaded on Mini-Protean^®^ TGX TM Precast gels 12% (BioRad, 456–1043) for 34 minutes at 200 V and 400 mA in Tris/Glycine/SDS Buffer, (BioRad, 1610732). Proteins were transferred onto 0.2 μm PVDF membranes (BioRad, 1704157) using Trans-Blot Turbo (BioRad) for 7 minutes using the following settings: 1.3 A, 25 V. Next, the membranes were blocked for 1 hour using Tris-buffered saline supplemented with 0.1% Tween 20% and 5% milk (Research Product International, M17200–500). Membranes were incubated with the primary antibodies: rabbit anti-MCT1/SLC16A1 pAb (Novus, NBP1–59656, lot C, 1:400), rabbit anti-MCT4 (Bethyl, A304–439A-M, 1:1000), rabbit mAb anti-beta-tubulin (9F3) (Cell Signaling, 2128S, 1:2000) diluted in Tris-buffered saline supplemented with 0.1% Tween 20% and 5% milk overnight. An anti-rabbit IgG HRP-linked secondary antibody (Cell Signaling, 7074S, 1:3000) was used to detect immune-reactive bands using enhanced chemiluminescence (ECL Western Blotting Substrate, Pierce, 32209) according to the manufacturer instructions. Quantification of protein bands was done by measuring band intensities using ImageJ software. MCT1 and MCT4 levels were normalized to beta-tubulin expression and expressed as the levels relative to the expression of Sham mice, which corresponds to 1.

### Machine Learning analyses

The ML pipeline is summarized in **Supplementary Fig. 3.** Preprocessing of the raw experimental data was performed before ML analyses to (i) scale the measurements for fair comparison and (ii) predict the missing measurements. The raw measurements were rescaled using Scikit-learn python library’s standard scaler^57^ (see **Supplementary Fig. 4** for an example of original versus scaled data distributions). For a few mice, we were not able to measure some of the variables due to tissue isolation (n = 2 rTBI and n = 2 Sham missing for PDH activity, n = 3–4 rTBI and n = 3 Sham missing for LDH activity, MCT1 and MCT4 expression) and low signal to noise of the HP [^13^C] imaging data (n = 1 rTBI mouse excluded). To impute the missing measurements we separated the data into rTBI and Sham groups, and within each group the data was split as training and testing. Mice with all measured variables were used to train a Random Forrest Regressor (RFR). The trained regressor was then used to predict missing measurements associated with the testing data.^58^ To identify the variables that are the best classifiers of Sham and rTBI groups, and to eliminate sensitivity of the results to different methods, we used multiple Scikit-learn classifiers and feature selection algorithims that are neural network, logistic regression, recursive feature elimination (RFE), SelectKBest, feature importance with ExtraTreesClassifier, and LASSO. Using each method, all the variables were scored based on their importance in the classification of Sham versus rTBI and a pool of variables with high importance scores was created. From this pool, we created groups of best classifiers with the minimum number of variables, that is three in this case, and ranked those triplets of variables based on their individual importance scores across different methods. Similarly, to identify the best predictors of the risk-taking behavior and HP ^13^C Lac/Pyr in the cortex, we trained multiple regression algorithms of Scikit-learn library that are LASSO, RFR, Ridge Regression, Support Vector Regressor, using the measurements of variable to be predicted as the y-values and all the remaining variables as the x-values (predictors). Then, the variables with high absolute coefficients were identified as the most important features that contribute to the prediction of the regressor. After pooling and grouping the variables with high feature scores across different algorithms, we observed the best predictor group with the minimum number of variables.

### Statistical analysis

Results are expressed as mean ± standard deviation (SD). Statistical analyses of MRI, behavioral, and ex vivo parameters was performed using unpaired t-test (GraphPad Prism (v 9.1.2), (*p ≤ 0.05, **p ≤ 0.01, ***p ≤ 0.001, ****p ≤ 0.0001).

## Figures and Tables

**Table 1:** PDH and LDH enzyme activity.

		PDH activity		LDH activity	
		mean ± SD	*P-value*	mean ± SD	*P-value*
**Prefrontal cortex**	rTBI	0.0007 ± 0.0001	**0.0044 (** ** **)**	0.0070 ± 0.0007	*0.3345*
	Sham	0.0011 ± 0.0003		0.0078 ± 0.0020	
**Cortex**	rTBI	0.0012 ± 0.0005	**0.0375 (** * **)**	0.0022 ± 0.0004	*0.5837*
	Sham	0.002 ± 0.0008		0.0023 ± 0.0003	
**Hippocampus**	rTBI	0.0074 ± 0.003	*0.4171*	0.0020 ± 0.0002	*0.1602*
	Sham	0.0091 ± 0.005		0.0023 ± 0.0005	
**Thalamus**	rTBI	0.00013 ± 0.00003	*0.6509*	0.0023 ± 0.0004	*0.1282*
	Sham	0.00014 ± 0.00006		0.0021 ± 0.0002	

Unpaired t-test.

* p ≤ 0.05

** p ≤ 0.01.

**Table 2:** MCT1 and MCT4 protein expression.

		MCT1		MCT4	
		mean ± SD	*P-value*	mean ± SD	*P-value*
**Prefrontal cortex**	rTBI	1.3 ± 0.31	*0.0685*	1.59 ± 0.72	*0.1109*
	Sham	1 ± 0.32		1 ± 0.54	
**Cortex**	rTBI	1 ± 0.14	*0.6399*	0.72 ± 0.35	*0.1502*
	Sham	1 ± 0.07		1 ± 0.33	
**Hippocampus**	rTBI	0.91 ± 0.31	*0.5090*	1 ± 0.77	*0.9147*
	Sham	1 ± 0.17		1 ± 0.58	
**Thalamus**	rTBI	0.89 ± 0.34	*0.4891*	0.75 ± 0.59	*0.3361*
	Sham	1 ± 0.24		1 ± 0.27	

Unpaired t-test.

**Table 3:** List of variables used for ML analyses.

Variable abbreviation	Variable description
EPM frequency extreme	Number of entries into the extreme zones on the EPM
EPM duration extreme	Time (sec) in the extreme zones on the EPM
EPM frequency openandcenter	Number of entries in the Open + Center on EPM
EPM duration openandcenter	Time (sec) in the open + Center on the EPM
EPM totaldistance centerpoint	Total distance traveled on EPM, measured by centerpoint, units = cm
EPM averagevelocity centerpoint	Average animal velocity on the EPM, units = cm/sec
HP ^13^C Urea Ctx	Hyperpolarized [^13^C]urea level in cortical area
HP ^13^C Lac/Pyr Ctx	Hyperpolarized lactate/pyruvate ratio in cortical area
HP ^13^C Urea Subctx	Hyperpolarized [^13^C]urea level in subcortical area
HP ^13^C Lac/Pyr Subctx	Hyperpolarized lactate/pyruvate ratio in subcortical area
PDH Pfc	PDH activity in prefrontal cortex
PDH Ctx	PDH activity in cortex
PDH Hp	PDH activity in hippocampus
PDH Thai	PDH activity in thalamus
nT_2_ Ctx	Normalized T_2_ intensity value in cortex
nT_2_ Hp	Normalized T_2_ intensity value in hippocampus
nT_2_ Pfc	Normalized T_2_ intensity value in prefrontal cortex
nT_2_ Subctx	Normalized T_2_ intensity value in subcortical areas
Volume Ctx	Volume of cortex calculted from T_2_w MRI
Volume Hp	Volume of hippocampus calculated from T_2_w MRI
Volume Pfc	Volume of prefrontal cortex calculated from T_2_w MRI
Volume Subctx	Volume of subcortical areas calculated from T_2_w MRI
Volume Ventricle	Volume of ventricles calculated from T_2_w MRI
Volume Brain	Volume of whole brain calculated from T_2_w MRI
T_1_ Ctx	T_1_ value in cortex
T_1_ Hp	T_1_ value in hippcampus
T_1_ Pfc	T_1_ value in prefrontal cortex
T_1_ Subctx	T_1_ value in subcortex
MCT1 Thal	MCT1 expression in thalamus
MCT1 Hp	MCT1 expression in hippocampus
MCT1 Ctx	MCT1 expression in cortex
MCT1 Pfc	MCT1 expression in prefrontal cortex
MCT4 Thal	MCT4 expression in thalamus
MCT4 Hp	MCT4 expression in hippocampus
MCT4 Ctx	MCT4 expression in cortex
MCT4 Pfc	MCT4 expression in prefrontal cortex
LDH Thal	LDH activity in thalamus
LDH Hp	LDH activity in hippocampus
LDH Ctx	LDH activity in cortex
LDH Pfc	LDH activity in prefrontal cortex
SWI Ctx	SWI value in cortex
SWI Hp	SWI value in hippcampus
SWI Pfc	SWI value in prefrontal cortex
SWI Subctx	SWI value in subcortex
